# Response to letter regarding long-term prognostic value of thyroid hormone levels in chronic critical illness patients

**DOI:** 10.1080/07853890.2025.2545552

**Published:** 2025-08-11

**Authors:** Zhaoxiang Li, Liang Wang, Yingjian Liang

**Affiliations:** ^a^ Department of Critical Care Medicine, The First Hospital of China Medical University, Shenyang, Liaoning Province, China; ^b^ Department of Critical Care Medicine, Tacheng District People’s Hospital, Tacheng, Xinjiang, China

Dear Editor

We thank Eeman et al. for the valuable comments in their letter to the editor regarding our published article ‘Long-term prognostic value of thyroid hormone levels in chronic critical illness patients’[1]. The letter focused on the timing of thyroid hormone testing and stratification based on comorbidities.

The course of the disease for critically ill patients is divided into two stages: the acute stage and the chronic critical stage. Some patients recover or die during the acute phase, while others survive during the acute phase and then enter the chronic critical illness stage [2]. Abnormal thyroid hormone levels can occur in the acute phase or in the chronic and severe stage, but the mechanisms of their occurrence are completely different [3]. During the acute phase, the changes in thyroid hormones are mainly due to alterations in thyroid hormone binding, peripheral thyroid hormone uptake, as well as changes in the expression and activity of type 1 and type 3 deiodinases. While in the chronic critical illness stage, the expression of thyrotropin-releasing hormone in the hypothalamus is inhibited, leading to a decrease in TSH secretion and thereby reducing the release of thyroid hormones. There have been numerous studies discussing the changes in thyroid hormone levels in patients during the acute phase and their impact on prognosis [4,5]. Our study focuses on the changes in thyroid hormone levels in patients with chronic critical illness and their impact on prognosis, so a longer observation period is needed.

NTIS is a clinical syndrome, caused by different etiologies and mechanisms. Different levels of thyroid hormones are also related to the severity of the disease, making it a heterogeneous disease. We agree with Dr Eeman’s view that using a stratified approach can provide a clearer understanding of the thyroid hormone patterns in different subgroups of people with chronic critical illness. Our next article is about the research in this aspect. Finally, we sincerely appreciate Eeman et al. for the interest in our article.

SincerelyYingjian Liang

## Data Availability

Data sharing is not applicable to this article as no data were created or analysed in this study.
